# Real Time Bayesian Estimation of the Epidemic Potential of Emerging Infectious Diseases

**DOI:** 10.1371/journal.pone.0002185

**Published:** 2008-05-14

**Authors:** Luís M. A. Bettencourt, Ruy M. Ribeiro

**Affiliations:** 1 Theoretical Division, Los Alamos National Laboratory, Los Alamos, New Mexico, United States of America; 2 Santa Fe Institute, Santa Fe, New Mexico, United States of America; University of Aberdeen, United Kingdom

## Abstract

**Background:**

Fast changes in human demographics worldwide, coupled with increased mobility, and modified land uses make the threat of emerging infectious diseases increasingly important. Currently there is worldwide alert for H5N1 avian influenza becoming as transmissible in humans as seasonal influenza, and potentially causing a pandemic of unprecedented proportions. Here we show how epidemiological surveillance data for emerging infectious diseases can be interpreted in real time to assess changes in transmissibility with quantified uncertainty, and to perform running time predictions of new cases and guide logistics allocations.

**Methodology/Principal Findings:**

We develop an extension of standard epidemiological models, appropriate for emerging infectious diseases, that describes the probabilistic progression of case numbers due to the concurrent effects of (incipient) human transmission and multiple introductions from a reservoir. The model is cast in terms of surveillance observables and immediately suggests a simple graphical estimation procedure for the effective reproductive number *R* (mean number of cases generated by an infectious individual) of standard epidemics. For emerging infectious diseases, which typically show large relative case number fluctuations over time, we develop a Bayesian scheme for real time estimation of the probability distribution of the effective reproduction number and show how to use such inferences to formulate significance tests on future epidemiological observations.

**Conclusions/Significance:**

Violations of these significance tests define statistical anomalies that may signal changes in the epidemiology of emerging diseases and should trigger further field investigation. We apply the methodology to case data from World Health Organization reports to place bounds on the current transmissibility of H5N1 influenza in humans and establish a statistical basis for monitoring its evolution in real time.

## Introduction

A pandemic of H5N1 influenza in birds is presently unfolding, with over 50 countries around the world affected, resulting in hundreds of millions of dead animals through infection or culling [1]–[3]. This emergency and the associated risk of a devastating new human pandemic [4]–[6] stress the need for new approaches targeted specifically at detecting and monitoring the evolution of *emerging* infectious diseases [7]–[9]. Assessing the risk of emergence of a human epidemic at the genetic level requires accounting for rare stochastic events, associated with genetic mutation and recombination, over vast pathogen and host populations [4], [8], [10]. This makes prediction of pathogenic evolution at the molecular level typically still very difficult. Consequently, the first indications of disease emergence are usually observed as infected cases in human and animal populations. Thus, for early assessments of the epidemic potential of a new outbreak, it is essential to assign quantitative meaning to existing epidemiological surveillance data in real time, with quantified uncertainty, and to use this knowledge to enable primary prevention strategies targeted at reducing chances of pathogenic evolution.

The quantity that measures the epidemic potential of a pathogen is the basic reproduction number *R*_0_ [11], [12]. *R*_0_ is defined as the average number of new infections created by an infectious individual in an entirely susceptible population. For established human pathogens, leading to standard epidemics, *R*_0_>1, as is the case of seasonal or pandemic influenza [13]–[19]. In practice, epidemiological data typically permit only the estimation of the *effective* reproduction number *R*, which may differ from *R*_0_ due to acquired immunity and other factors. For an emerging infectious disease, when transmission is only incipient [20] and the pathogen is adapting to the population, it becomes crucial to monitor quantitative changes of the effective reproduction number over time. Thus, the detection and tracking of an emerging disease can be formalized in terms of monitoring *R*, as it evolves and approaches the critical threshold *R*→1. This is likely the current state of H5N1 avian influenza in humans, where complete absence of human to human transmission would imply *R* = 0, but likely *R* is very small, as a few cases of possible human contagion suggest [21]–[23].

**Figure 1:**
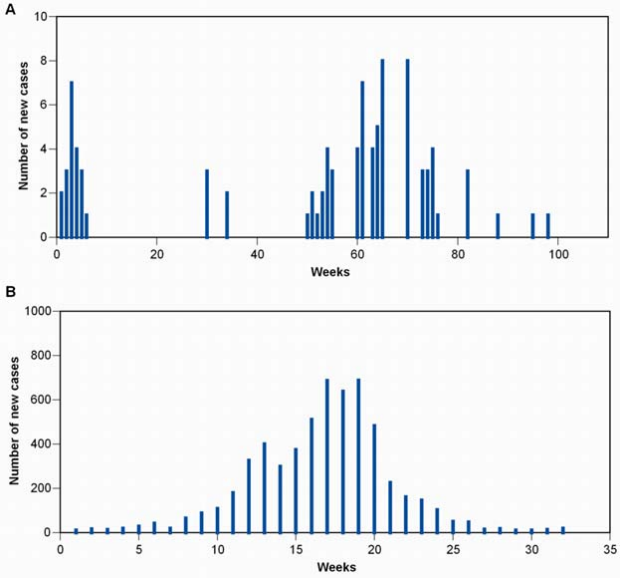
Time series of new cases for an emerging infectious disease *vs.* a standard epidemic. (a) Laboratory confirmed new human H5N1 avian influenza cases, from WHO reports in Vietnam (from January 2004 to June 2006); (b) Number of isolates for seasonal H3N2 influenza in the USA, over the 2004–2005 season. Note the 100-fold difference in case numbers (*y*-axis) between panel (a) and (b). For an emerging infectious disease such as H5N1 influenza in humans, case numbers are small, very stochastic, and alternate short outbreaks with long quiet periods.

Notwithstanding a marked recent increase in systematic surveillance by national and international organizations, and the advent of real time reporting of many public health indicators (syndromics) [24], the epidemiological regime of incipient but evolving transmission has received little attention in terms of quantitative modelling [22], [25]–[28]. The main difficulty is that data in these circumstances tend to be very stochastic, involve small case numbers and may be plagued by uncertainties and inconsistent reporting. As an example, we contrast in Figure 1 the time series of confirmed new human cases of H5N1 avian influenza in Vietnam, reported by the World Health Organization (WHO), with weekly isolate numbers for seasonal H3N2 influenza in the USA during 2004–2005 (see Methods for “Data Sources”). The ultimate objective of this paper is to propose a methodology to extract quantitative inferences and generate epidemiological outlook in real time from time series like that of Figure 1a.

Recently the problem of real time monitoring of (emerging) communicable diseases has gained growing attention, with a few new methods proposed to estimate *R*. One method proposes the analysis of the distribution of the sizes of case clusters to provide indications of changes in *R*. Specifically, increases in *R*(<1) translate on average into larger case cluster sizes [21], [27]–[29]. Another approach [30] relies on the inference of probable chains of transmission among observed cases from knowledge of the statistical distribution of the infectious period. From an ensemble of such chains and their associate compounded probability, *R* can be estimated. This method has recently been applied to “real time” monitoring of SARS [31], [32], via a Bayesian inference scheme. The strength of this class of methods is that they allow insights into heterogeneities in the population. This demands the consideration of all pairs of possible transmissions, which may become computationally intense as case numbers rise and can be sensitive to under reporting, competing risk and to the details of the distribution of infectious periods. Moreover those studies considered the efficacy of control measures for a disease with an initial *R*>1 and no new cases introduced during the epidemic, whereas it is typical of emerging communicable diseases that adaptation of the pathogen's tropism to the host population is the result of numerous such introductions [22].

Here we propose an alternative approach, which addresses the issue of new introductions, requires in general smaller computational overhead and results in the estimation of the full probability distribution for *R*. The method is based on the probabilistic formulation of standard SIR disease transmission models analogous to the time-series SIR (TSIR) approach [33], which simplifies the need to reconstruct transmission chains by aggregating all infectious and susceptible individuals into classes that are assumed to mix homogeneously. A Bayesian procedure is then employed to translate the time series of case numbers into a probability distribution for epidemiological parameters. The method adopts the standard assumptions made in epidemiological compartment models with homogeneously mixing classes, and benefits from their simpler computational structure allowing efficient estimation with available sparse empirical data. The estimation method developed here has been applied once before [34] to 1918 influenza pandemic death notifications time series for San Francisco, with the purpose of comparing its performance with other conventional methods for estimating *R*. Here we present its full derivation, provide more details and examples, include introductions from an animal reservoir and show how the method can be used to provide statistical expectations for new case predictions. We also show how case predictions with quantified uncertainty do, in turn, define possible statistical anomalies for future case numbers, which can be used to inform surveillance and logistical management in the event of a new or continuing outbreak. As an example, we apply the method to human case data of H5N1 influenza in Vietnam and Indonesia, to produce bounds on its effective reproduction number, R, and establish a basis for its continued monitoring in real time.

## Materials and Methods

### Data sources

Time series of H5N1 influenza cases in humans were assembled from World Health Organization (WHO) reports of confirmed cases (http://www.who.int/csr/don/en/), from January 2004 to June 1, 2006 (see Supplementary Material S1, including Figure S1, for more information). Data for H3N2 seasonal influenza was obtained from the Centre for Disease Control (CDC) Surveillance Weekly Reports in the United States (http://www.cdc.gov/flu/weekly/fluactivity.htm).

### Model development

New human cases of avian influenza may result from two alternative processes: i) infection of humans from animal sources [4], or ii) human to human transmission [23]. For a standard epidemic, explicit consideration of multiple introductions is not important as each case produces many secondary infections. For emerging infectious diseases multiple introductions from a reservoir [22], [25] may constitute an important fraction of all observed cases, and the progression of secondary cases must be carefully assessed and monitored.

Our objective is to cast standard SIR-class models in a form that directly relates to time series data of emerging infectious diseases by *i*) accounting for cases from reservoir sources, *ii*) casting the model variables in terms of observable quantities reported from field surveillance, *iii*) formulating the model in a discrete probabilistic form, and *iv*) quantifying uncertainty in the estimation of epidemiological parameters and future cases, and assimilate new data to reduce it.

### Average case progression in the absence of multiple introductions

We consider a standard epidemic susceptible-infected (SIR) model
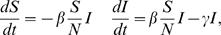
(1)where *S*(*t*) is the average number of susceptibles at time *t*, *I*(*t*) is the average number of infectious, *N* is the size of the population, which decreases due to disease-induced deaths (taken as a fraction α of progressing infections), β is the contact rate, and γ^−1^ is the infectious period. After an average residence time γ^−1^, infectious individuals recover or die (not shown in [1]). The total number of cases up to time *t*, *T*(*t*) obeys the equation *dT*/*dt* = β *S*/*N I*. Epidemic reports most commonly state the occurrence of new infected cases, which over the period τ, are given by *T*(*t*+τ)−*T*(*t*) = Δ*T*(*t*+τ).

To find the expression accounting for the evolution of new cases Δ*T*(*t*+τ) we integrate Eq. [1], for *I*(*t*) between *t* and *t*+τ, to obtain
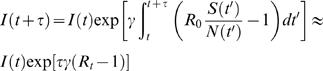
(2)where *R*_0_ = β/γ, and *R_t_* = (*S*(*t*)/*N*(*t*))×*R*_0_ (the “instantaneous” reproduction number) is a function of time; the last expression is exact if *S*(*t*)/*N*(*t*) is constant in the period [*t*, *t*+τ]. This simplifying assumption is generally excellent for emerging infectious diseases, which result in few cases within a much larger population. Generally the validity of the assumption can be assessed through consideration, from [1], of its evolution equation
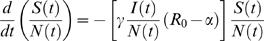
(3)which shows that *S*(*t*)/*N*(*t*) is approximately constant over a time interval τ, if τγ*I*(*t*)/*N*(*t*)(*R*_0_−α)<<1. This condition is usually satisfied as the fraction of infectious at a given time, *I*(*t*)/*N*(*t*), is typically less than a few percent (even for seasonal influenza), while other quantities in the product are of order unity. The quantity, in expression [2], 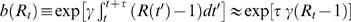 evolves *I*(*t*) to *I*(*t*+τ), accounting for the number of new cases resulting from infections over time τ [35].

To obtain the disease progression in terms of epidemiological observables, we discretize the differential equation for the change in total number of cases between *t* and *t*+τ as
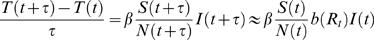
(4)where we used [2] and the assumption that *S*(*t*)/*N*(*t*) is piecewise constant over [*t*, *t*+τ], but does vary between intervals contributing to changes in *R_t_*. At time *t*, the total number of cases is also
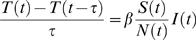
(5)


Substituting expression [5] into [4], we obtain:
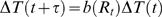
(6)


**Figure 2:**
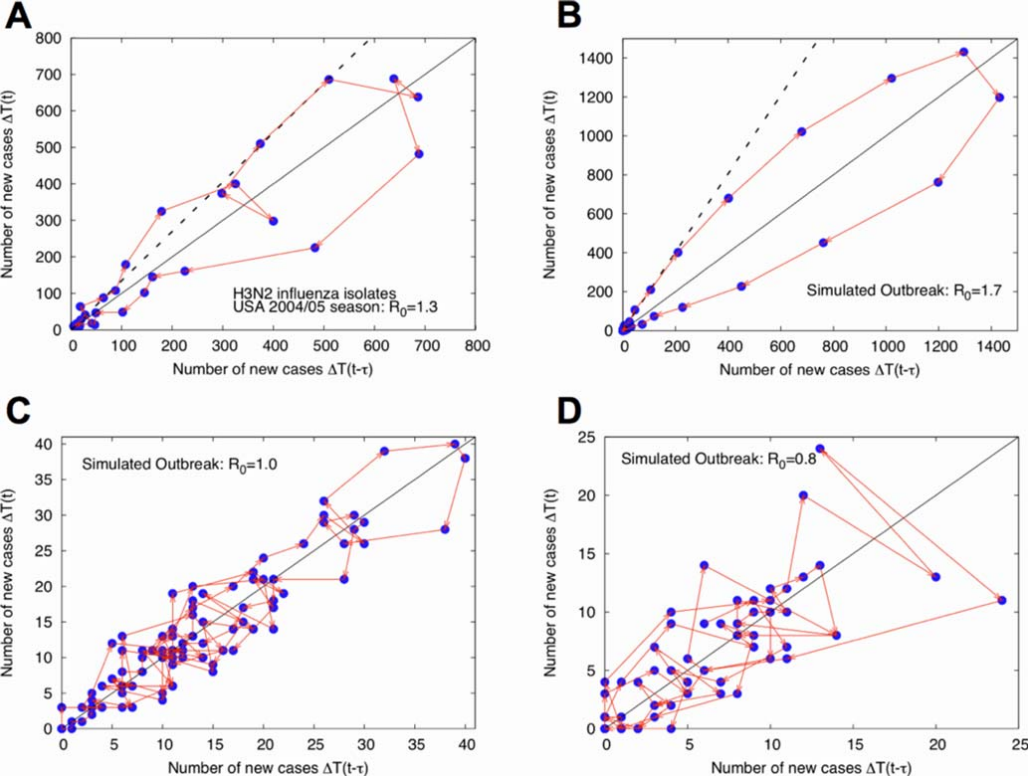
Epidemic time delay diagrams for different *R*_0_. (a) Relation between new cases at consecutive time periods (weeks) for H3N2 isolates in the US 2004–05 season, and for simulated data with (b) *R*_0_ = 1.7, (c) *R*_0_ = 1.0 and (d) *R*_0_ = 0.8. For these simulations, the introduction of new cases from the reservoir follows the Vietnam case history, Figure 1a. New cases are then generated using expression [11], according to a Poisson distribution. The trajectories connecting new cases at consecutive times (red arrows) eventually return to the origin because depletion of susceptibles reduces the *effective* reproduction number (i.e. the *actual* number of secondary cases produced by an infectious individual). Dashed lines in (a) and (b) are the tangents at the origin to the case number trajectories (red arrows), with slope *b*(*R*).

We see that the well known multiplicative progression between new cases at successive times due to contagion appears, on average, as a linear relation between Δ*T*(*t*+τ) and Δ*T*(*t*) in an epidemic time delay diagram, Figures 2a–d. Expression [6] generalizes similar relations in the TSIR literature by casting them in terms of new cases over arbitrarily chosen observation intervals τ, not necessarily coinciding with the average generation time γ^−1^. Expression [6] also shows how the initial *R_t_* can be estimated geometrically (without the need for parameter search or numerical optimization) from an epidemic time delay plot of surveillance data: *b*(*R_t_*) is the slope of the tangent at the origin of case trajectories (dashed line in Fig. 2a, b). For emerging infectious diseases relative fluctuations in case numbers are large, see e.g. Figure 2d, and this simple geometric approach is not valid, thus making more robust estimation methods, as the one presented here, necessary.

### Progression of new cases due to human contagion and multiple introductions

For emerging infectious diseases, many introductions from a reservoir may occur before the pathogen adapts its tropism to the new host population and produces epidemic outbreaks [22], [25]. As a result epidemiological models for the time evolution of new cases must account for two processes: (incipient) human transmission and infections from the reservoir.

We introduce a new source of infected individuals, through contact with the reservoir (birds). The evolution of *I* is now given by
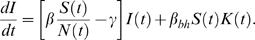
(7)


The first term on the right accounts for the human-to-human infectious process. The last term is a source, creating new *I* through contact with a reservoir of infectious agents of size *K*(*t*), with contact rate β_bh_.

We denote the number of new infections from the reservoir per unit time as *dB*/*dt* = β_bh_ *S*(*t*) *K*(*t*). As a result the number of humans infectious, *I*, and the total number of cases evolve as
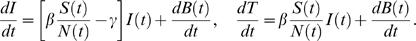
(8)


The evolution of *I*(*t*) between *t* and *t*+τ, accounting for the effects of the inhomogeneous source term, is
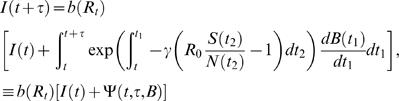
(9)where Ψ(*t*, τ ,*B*) denotes the integral. We use this expression to solve for the number of *new* cases (Eq. [8]), giving
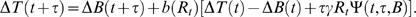
(10)


### Probabilistic models of contagion

A probabilistic description is crucial for realistic modelling of new cases of emerging infectious diseases, which are typically characterized by large coefficients of variation. This probabilistic description is achieved, as in [33], by defining the number of new cases, Δ*T*(*t*+τ) as a stochastic discrete variable generated by a probability distribution with average number of cases given by [10], i.e.
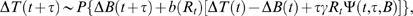
(11)where *P*{λ} denotes a discrete probability distribution with mean λ. If the only information on case evolution is their average number then the maximum entropy distribution for *P*{} is Poisson, which we adopt throughout this paper. If additional information on the magnitude of fluctuations were also known, a generalized distribution should be employed, such as a negative binomial [33], which can account for clumping effects (we reproduce all estimations from the main paper using a negative binomial in Supplementary Material S1, Table S2).

To use expression [11] in practice, we need to evaluate the integral Ψ. Assuming the introductions from the reservoir per unit time to be approximately constant between *t* and *t*+τ, the integral can be calculated to first order as Ψ(*t*,τ,*B*) = τ *dB*/*dt* and, [11] is written as
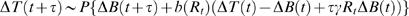
(12)where we replaced τ*dB*/*dt* by its discrete approximation Δ*B*(*t*). This is the expression used in practice in all quantitative estimations presented.

### Bayesian estimation of *R* with quantified uncertainty

Parameter estimation with quantified uncertainty can be achieved using a Bayesian approach in the context of probabilistic epidemiological models. Bayes' theorem expresses the full probability distribution for model parameters, such as the effective reproduction number, *R*, in terms of the probabilistic epidemiological model [12], given the time series for new cases. Specifically, the probability distribution of *R*, compatible with the observed temporal data stream is given by
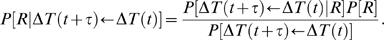
(13)


*P*[*R*] is the *prior*, which captures given knowledge of the distribution of *R*. The distribution *P*[Δ*T*(*t*+τ)←Δ*T*(*t*)] is independent of *R*, and corresponds to a trivial normalization. From successive applications of Bayes' theorem, a sequential estimation scheme, that uses streaming epidemiological observations performed in real time, can be constructed using the posterior distribution for *R*, at time *t* as the prior in the next estimation step at time *t*+τ, leading to an update scheme via iteration of Eq. [13]. The resulting probability distribution for *R* includes information on all observations up to time *t*, and contrasts with the “instantaneous” *R_t_*, used above, which only considers the data at times *t* and *t*+τ. Thus, *R* is a robust estimator of the effective reproduction number assumed to be constant for the whole epidemic up to time *t*. Any changes in *R* over time result from the assimilation of each new data point, leading to an updated estimate of *R*. This in turn allows the use of our estimation procedure as an anomaly detection tool (see below).

### Time series of introductions and contagion

Ideally, field case tracing should provide a measure of the likelihood that a new case resulted from contact with the (animal) reservoir, Δ*B* in Eq. [12], or was due instead to human contagion. Another possibility is to explicitly model the introductions from the reservoir, *K*(t) in Eq. [7]. Although some empirical studies start to address this possibility [27], [36]–[38], it is still difficult to calibrate such models and uncertainties remain large. Thus, for the calculations in this study, we choose to use a minimal statistical approach. Formally, assuming statistical independence between different cases, we model each new introduction as a Bernoulli trial with probability θ. θ is defined as the average probability that a case is attributed to human to human contagion, and 1-θ that it is the result of an infection from the reservoir. Note that for emerging infectious diseases this probabilistic model is more appropriate and generalizes (see Supplementary Material S1, Figure S2) the more typical modelling of introductions as homogeneous Poisson processes [33].

We can provide an upper bound for θ by considering observed clusters of cases. If we take all confirmed cases in clusters, except the index case, to be due to human contagion, then an estimate for θ is given by the proportion of such cases divided by the total number observed over the same period. This gives an upper bound on θ, because it is unlikely that all cluster cases arise from human infection, rather some could have a common reservoir source. Two epidemiological studies of H5N1 influenza, one from January 2004 to July 2005 [39] and another from July 2005 to June 2006 [40], found that 26 of 109 cases and 15 of 54 cases, respectively, occurred in family clusters Attributing those cases to human infection gives θ = 0.24–0.28. This estimate of θ is consistent with an independent statistical analysis of a case cluster in Indonesia, which found that the secondary attack rate for household transmission of H5N1 influenza was 0.29 [28].

In the remaining of this paper we treat θ as a constant parameter common to all reported cases. The sensitivity of *R* estimates is then assessed as a function of θ (Table 1). In addition to tests of the estimation procedure on epidemic data [34], we also verified the precision of our methodology on an extensive number of simulated case time series, based on a standard SIR model, with introductions from a reservoir and different *R*_0_.

**Table 1:** Bounds on R for different probabilities of human transmission, θ.

	VIETNAM				INDONESIA			
	Average fraction of cases attributable to human contagion (θ)							
	1.0	0.8	0.29	0.2	1.0	0.8	0.29	0.2
***R*** ** min**	0.26	0.23	0	0	0.16	0.09	0	0
**ML ** ***R***	0.53	0.46	0	0	0.56	0.43	0	0
**Mean ** ***R***	0.52	0.46	0.25	0	0.54	0.42	0	0
***R*** ** max**	0.77	0.68	0.42	0	0.89	0.75	0	0

Current estimates of *R* (*i.e.* highest probability *R* – ML *R*; and posterior mean *R*) for human H5N1 avian influenza, obtained from new case time series for Vietnam and Indonesia. “*R* min” and “*R* max” denote the lower and upper bounds of the 95% credible intervals, respectively. θ is the probability of human-to-human transmission (see Methods). Analysis of reported cases from Thailand, China, and Turkey lead to similar or lower *R* estimates, but display wider bounds due to smaller case numbers.

### Numerical parameter estimation

We used the observed time series of new cases of human H5N1 influenza Δ*T*(*t*) to compute the probability distribution for *R* using programs implemented both in Matlab and Fortran. We used an unbiased uniform distribution for *R* between 0 and 3 as the initial prior. For each subsequent weekly iteration, we computed the full posterior distribution from [13] using the posterior at the previous week as the new prior. The product of the two probabilities on the right-hand side of [13] was evaluated as a non-parametric function defined in terms of 1000 discrete bins in *R* between 0 and 3, as shown in Figure S3 in the Supplementary Material S1. Parameters choices used in the calculations in the main text are: τ = 1 week, γ = 1 week^−1^, θ variable as in Table 1. We also explored other parameter choices reported in the literature for seasonal influenza and corresponding results are given in Table S1 of Supplementary Material S1.

## Results

### Simulated Outbreaks

Here we show how the method performs at estimating *R* from single realization time series, produced by simulation with a known value of *R*_0_. In all instances the time series for human H5N1 cases in Vietnam (Figure 1a) was used as introductions into the human population. For a choice of *R*_0_>1 in the simulation, any introduction readily develops into an epidemic. For *R*_0_<1 each introduction leads to small outbreaks that eventually become extinguished.

**Figure 3:**
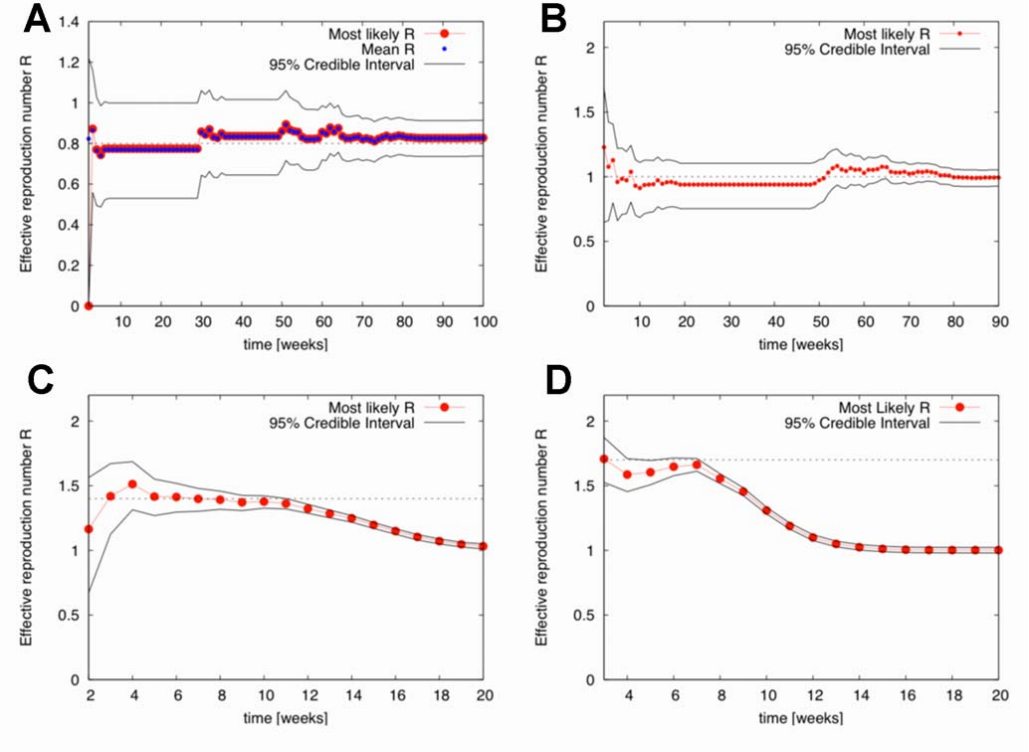
Evolution of *R* estimates over time (weeks) for single realization simulated data with *R*_0_ = 0.8, 1.0, 1.4 and 1.7 (left to right, top to bottom). Dashed lines indicate the value of *R*_0_ in the simulation. The decay of *R* estimates over time in standard epidemics is due to the depletion of susceptibles. For *R*_0_ = 1.0, 1.4 and 1.7 the mean is indistinguishable from the estimate of *R* with maximum probability and is not shown.

The effective reproduction number, *R*, calculated by our method changes over time, because of decreases in the fraction of susceptibles, *S*(*t*)/*N*(*t*), and the availability of more information, as more cases are observed. Thus, we use the values obtained for *R* at early times, when *S*(*t*)/*N*(*t*) approximates its initial value, to estimate *R*_0_ of the simulation by assuming that max(*R*) = *R*_0_. As shown in Figure 3, in all circumstances, the method gives an excellent estimation of *R*_0_ as outbreaks unfold, usually making accurate predictions when supplied with a mere two or three observation points. Uncertainty, measured by the width of the 95% credible interval, is reduced by larger case numbers, so that it typically remains higher the smaller the *R*_0_. In all instances uncertainty is reduced as more cases are reported over time.

### Bounds on R for avian influenza in humans from WHO reported time series

We next applied our method to the time series of cases of H5N1 influenza in humans. We produce estimates and credible bounds for *R*, under different scenarios for the expected fraction of new observed human cases that is attributable to human contagion θ. Summary results for Vietnam (and Indonesia) are given in Table 1. Even in the worst case scenario, where all observed cases are attributed to human transmission (θ = 1), the most likely (as of June 2006) *R* is 0.53 (0.56), with an upper 95% bound of *R*<0.77 (0.89). For the estimated θ = 0.29 (see Methods), the most likely *R* for both Vietnam and Indonesia is 0, although the estimated upper 95% bound in Vietnam gives the bound *R*<0.42. For Indonesia, the corresponding estimate gives an *R* entirely consistent with zero at the 95% credible level.

For less than 20% of the cases attributable to human transmission, *R* is entirely consistent with zero, even when accounting for the uncertainty in the duration of the infectious period (γ^−1^). Reported information does not allow at present a precise determination of γ^−1^ for H5N1 influenza in humans, so that different scenarios are possible, which we explore in detail in Supplementary Material S1 (Figure S4, Table S1). Data permitting, a hierarchical Bayesian estimation method for the distribution of γ can also be envisaged [32].

**Figure 4:**
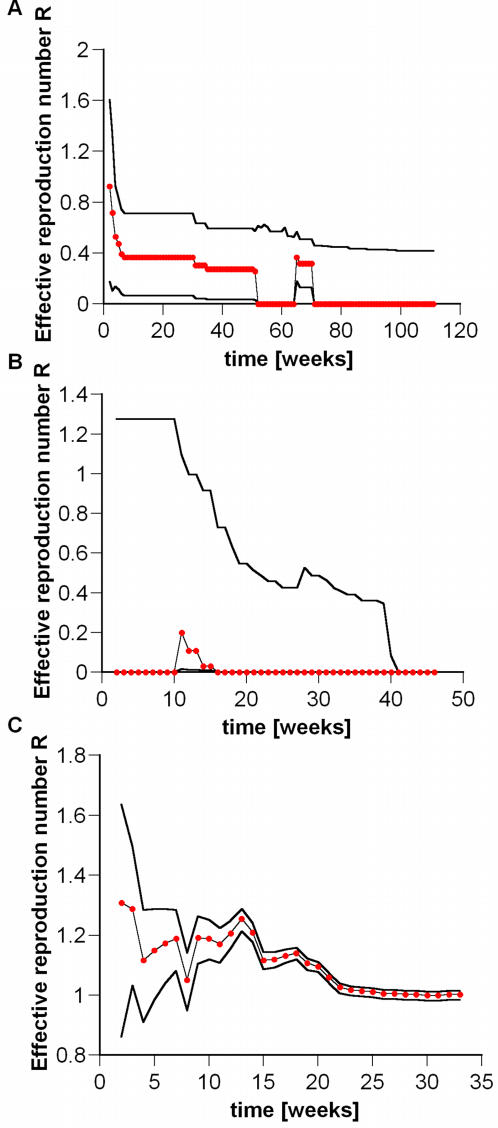
Sequential Bayesian estimation of the posterior mean *R* (red dots) and 95% credible intervals (solid lines) for the time series of H5N1 avian influenza in (a) Vietnam and (b) Indonesia, under the pessimistic assumption that 29% of reported cases are due to human-to-human transmission (see Table 1); and (c) for seasonal H3N2 human influenza isolates in the USA during the 2004–2005 season. (Note that isolates represent only a small fraction of total cases, and may contain reporting biases.) The estimate of the effective reproduction number for an epidemic outbreak asymptotes to unity at late times because initial growth and long-term decay in new case numbers (due to depletion of susceptibles) average out over the history of the outbreak.

Figure 4 shows the evolution of *R* and of its corresponding 95% credible interval. The computation of successive probability distributions for *R* gives a basis for assessing the evolution of transmissibility over time, including the approach to the epidemic threshold *R*→1. At present we conclude that, even in the unrealistic worst case scenario, where cases are aggregated at the national level and all cases are attributed to human transmission, *R* remains below unity.

### Shifts in transmissibility as statistical anomalies

**Figure 5:**
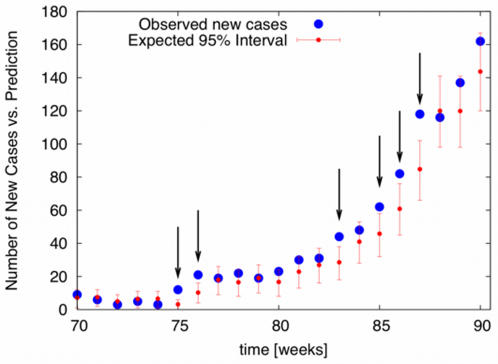
Prediction for new cases of avian influenza (simulated data *R*_0_ = 0.8, with infections from reservoir taken from Vietnam time series, Fig. 1a) *vs.* realized new cases (blue dots). Between weeks 74 and 75, the reproduction number is shifted *R*_0_ = 0.8→1.3 to create an epidemic. Although we continued to iterate the *R* distributions via the Bayesian procedure described in the text, note that the shift in *R* upwards leads to many statistical anomalies (indicated by black arrows). The anomaly is detected immediately, on weeks 75 and 76. Anomalies here are defined as observed numbers of new cases that fall outside the expected 95% credible interval. These anomalies indicate a violation of the hypothesis that *R* is unchanged, and could be used to trigger alerts in surveillance.

The emergence of a new epidemic in humans often requires shifts in pathogen biology and/or changes in the human population structure. The methodology developed here can signal these events as anomalies in the expected number of new cases. Assuming no change of epidemiological conditions, knowledge of the distribution of *R*, accumulated until time *t*, provides expectations for future case numbers Δ*T*(*t*+τ) , with quantified credible intervals, via
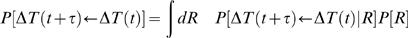
(14)where *P*[*R*] is taken as the posterior in [13] at time *t*, and *P*[Δ*T*(*t*+τ)←Δ*T*(*t*)|*R*] is the statistical epidemic model. Failure to predict future observed cases at time *t*+τ, can then be formulated as a p-value significance test at any chosen level of credibility. A statistical anomaly, *i.e.*, future cases falling outside the credible interval defined by previous observations, may signal changes in epidemiological parameters, specifically in transmissibility (either by pathogen evolution or host population changes) as measured by *R*. We provide an example in Figure 5, for simulated data with *R*_0_ = 0.8 changing to *R*_0_ = 1.3, where we show the predicted 95% credible interval for new cases *vs.* the number of cases actually observed (see also Figure S7 in Supplementary Material S1).

## Discussion

Emerging and re-emerging infectious diseases pose some of the greatest health risks to human populations worldwide. Increasingly they are a feature of our time, stoked by changes in human demographics, mobility, land use and climate, and compounded by poor standards of public health in parts of the world [26], [41]. Importantly, new surveillance and intervention strategies are now becoming possible, guided by quantitative interpretation of epidemiological data, potentially strengthening the hand of primary prevention efforts.

The modelling and prediction approaches developed here (see also Chowell et al. [34] for a comparison to other methods) provide tools for real time estimation of epidemiological parameters that are appropriate for emerging infectious diseases. The method is intentionally simple, relying on standard epidemiological population models, in order to be commensurate with the paucity of epidemiological data typically available for emerging infectious diseases. These features are illustrated by the application of the method to H5N1 influenza infection time series in humans. Clearly, the SIR class of models, even when cast in probabilistic terms, relies on several general assumptions, which are simplistic in specific situations. First, these models do not account for contact heterogeneities, resulting from spatial effects, age, and/or the structure of social networks. These effects can be partially addressed by structuring the population compartments in terms of spatial and risk classes [42]. For example, different regions in an affected country may be taken as separate compartments, provided that there are data pertaining to each region. Indeed the method would then allow estimation of correlations between *R* at different spatial locations. Second, in its present form, the model does not include independent estimation of infectious or incubation periods. It is straightforward to include an incubation period [43] and, given data on the duration of these periods on a case by case basis, these issues can be addressed by including additional Bayesian estimation steps (see [32]). Notwithstanding these limiting features, the SIR structure allows reliable real time parameter estimation with quantified uncertainty at very low computational overhead, as verified extensively via simulations at varying known input *R*, and applications to past pandemic outbreaks (Supplementary Material S1 – Fig. S5 and S6 – and Ref [34]).

One feature of the bounds on *R* derived here is their dependency on the fraction of cases attributed to human transmission, θ. Although θ is judged to be small from present surveillance [23], [27], [28], it remains hard to quantify with certainty. Given the paucity of data, we chose in practice to assume independence and use a binomial probability to attribute cases to human transmission vs. infection from the reservoir. However, other procedures to determine θ are possible. If enough data were available, an explicit dynamical model of the (animal) reservoir could be built, or an empirical function correlating introductions through time could be used. Indeed, in the optimal scenario, the actual cases of introduction from the reservoir would be known from field work, and the method proposed here could incorporate that information directly. We note that in the most relevant case, when *R* becomes larger than 1, the effect of θ<1 quickly vanishes, as cases multiply exponentially. For R<1, even a choice of θ = 1 will lead to estimates of R<1, but different values of θ may lead to credible intervals that include the critical threshold. In general, we believe that a suspicion of a possible *R*≈1 should be followed up with careful field investigations.

We presented a general methodology capable of interpreting quantitatively emerging disease surveillance data in real time with quantified uncertainty that complements other methods proposed recently [21], [29], [30], [32]. Although we illustrated the method with data for H5N1 influenza, these inference strategies are general and can be applied to time series from other communicable diseases. We verified that the model developed here also applies to standard epidemics, yielding agreement with previous estimates of *R* for the 1918 influenza pandemic in US cities [14], [44] (Figures S5, S6 in Supporting Information) and with other estimation methods [34]. We have also shown how to construct p-value statistical significance tests suitable for automatically monitoring changes in transmissibility of (emerging) communicable diseases [43].

While still in their infancy, we believe that the current emerging trend in mathematical epidemiology towards real time predictive methods will enable a shift towards more quantitative surveillance and primary prevention, resulting in more consistent and extensive monitoring of emerging infectious diseases and improved designs for health interventions and logistic allocations as epidemics unfold.

## Supporting Information

Material S1 In Supplementary Material S1, we present further details of the method, including extensions and additional examples.
(0.28 MB PDF)
