# Supplementary material for: Real Time Bayesian Estimation of the Epidemic Potential of Emerging Infectious Diseases
Source: PLoS One. 2008 May 14;3(5):e2185. doi: 10.1371/journal.pone.0002185 (PMC2366072; doi:10.1371/journal.pone.0002185)
Supplement: Material S1 — In Supplementary Material S1, we present further details of the method, including extensions and additional examples. (0.28 MB PDF) [file pone.0002185.s001.pdf]

Supplementary Material S1:
Real time Bayesian estimation of the epidemic potential of emerging infectious diseases

1 Time between onset of symptoms and death for confirmed cases of H5N1 influenza in humans

Case information on the nation, region, date of onset of symptoms/disease, hospitalization, death/survival and time of death were compiled from World Health Organization "situation update" reports. Some or most of these fields are not reported in many of the updates, especially the epidemiologically important dates of onset of symptoms and clinical recovery or death. Figure S1 shows the histogram of the time between onset of symptoms and death for all cases (number of observations n=44) for which this information is available. Note that Figure S1 bears a close resemblance to Supplementary Information Figure 1 of [1], for the 1918 H1N1 pandemic influenza (n=94). Also, for these cases, which correspond to hospitalizations in most cases, the mode of the time between onset of symptoms and death is about 9 days.

2 Temporal statistics of cases and introductions

We calculated the average number of cases per week ($\lambda = 0.73$) from reported cases, and verified that case introductions of avian influenza, taken as a fixed large fraction of cases, are not well modelled by a homogeneous Poisson process with mean λ (Figure S2).

This has important consequences that guided our statistical modeling of introductions. First, we

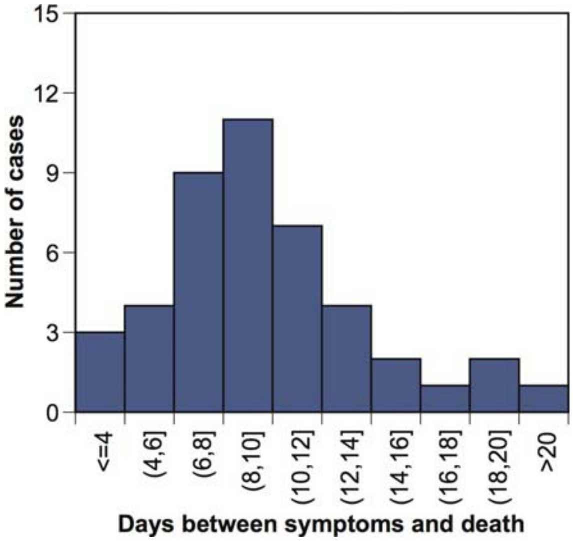

Figure S1: Histogram of times between onset of symptoms and death for cases of H5N1 influenza in humans worldwide. Compare to Figure 1 in Supplementary Information of [1] for similar numbers regarding the 1918 H1N1 influenza pandemic.

want to emphasize that the probabilistic modeling of introductions is necessary and standard in general (given that definite knowledge of the processes that start infection clusters are almost always unknown). For example, in [6] it is assumed that the temporal structure of infectious immigrations follows a homogeneous Poisson distribution with a constant rate. However, for the purposes of modeling emerging infectious diseases with a small number of cases, this is not an appropriate choice. There are two reasons for this, one formal and one empirical:

Formally, there is a crucial difference in the context of emerging infections (with effective $R < 1$): there is a very small number of cases, which forces the modeling of introductions with a risk of infection per case θ to be done in terms of Bernoulli trials (binomial distribution), as we propose in methods. A Poisson model may approximate this process over many case numbers n, in the large numbers limit where n becomes large but the average number of introductions, $\lambda = n \times p$, stays constant.

Empirically, the use of a homogeneous Poisson model produces nonsensical data because of the fact that a constant risk of infection often leads to more cases being predicted than observed, thus sometimes implying an incorrect negative number of human-to-human infections.

Ultimately, the best solution is for the probability of introductions to be estimated from case tracing information. Unfortunately, however, quantitative information of θ is absent for the vast majority of reported cases of H5N1 influenza. Consequently, for estimation purposes, we chose a simple strategy to set a value for θ common to all cases and vary it in order to produce bounds on R under different scenarios. We emphasize that we used a constant θ for conceptual and practical simplicity, but that the method is designed to accommodate θ varying from case to case if known, or even to eliminate it completely as a statistical process, if there is certain knowledge of the number of infections resulting from the reservoir.

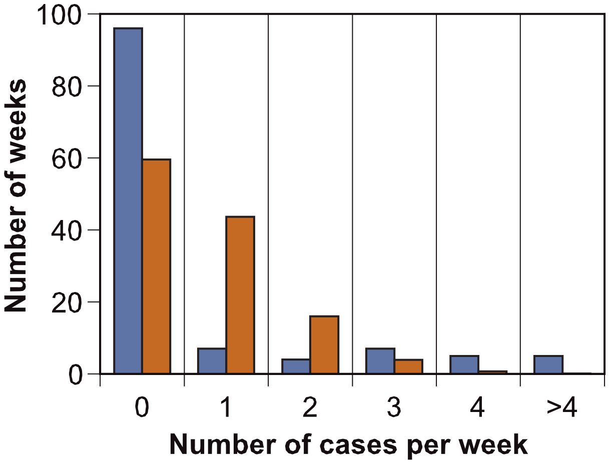

Figure S2: Comparison of the observed distribution of the number of cases per week in Vietnam (blue) and a Poisson distribution with the same mean ($\lambda = 0.73$) (red).

3 Evolution of the probability distribution for R via iterative Bayesian estimation

The method proposed in the main text produces an estimate for the full distribution of the effective reproduction number. R estimated in this way takes into consideration all observations up to the current time. R varies in time and it can be shown that at the end of a full epidemic cycle $R = 1$. This should not be confused with what we denoted the ”instantaneous” reproduction number, R_t, in the main text, which only takes into consideration data at time t and $t + \tau$. Indeed, it is clear that towards the end of an epidemic cycle $R_t < 1$ due to the depletion of susceptibles. In our study, we are concerned with and present estimates of R. Figure S3, shows an example of the outcome of the method for the evolution of the probability of the effective reproduction number, R.

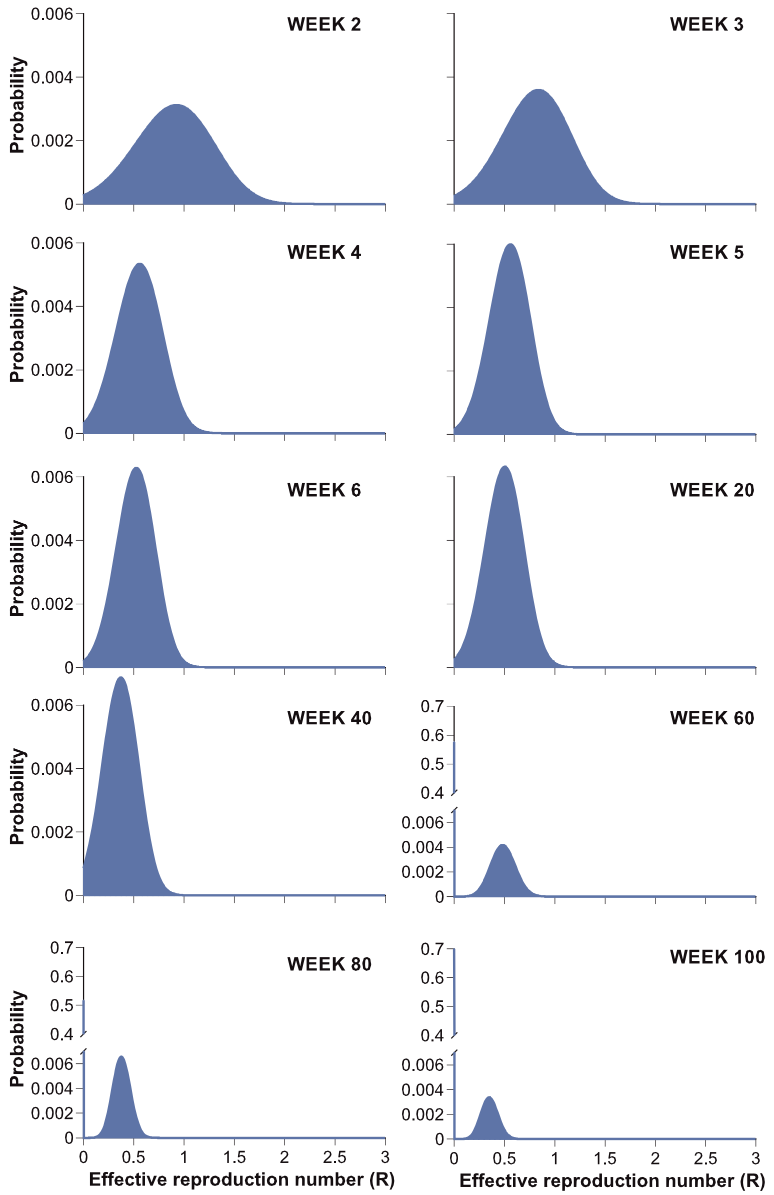

Figure S3: Time evolution of the full probability distribution of R over time, obtained via the sequential iterative estimation described in the main text, for the time series of human H5N1 influenza cases in Vietnam, Figure 1a (main text).

Infectious period (days)	θ=1.0	θ=0.8	θ=0.6	θ=0.4	θ=0.2
2.6	0.83<br>0.83<br>(0.72,0.92)	0.70<br>0.70<br>(0.60,0.78)	0.59<br>0.59<br>(0.49,0.68)	0.49<br>0.49<br>(0.39,0.58)	0<br>0.14<br>(0,0.43)
4.1	0.73<br>0.72<br>(0.56,0.87)	0.61<br>0.60<br>(0.46,0.74)	0.51<br>0.51<br>(0.37,0.63)	0.41<br>0.41<br>(0.28,0.53)	0<br>0<br>(0,0)
7.0	0.53<br>0.52<br>(0.26,0.77)	0.46<br>0.46<br>(0.23,0.68)	0.40<br>0.40<br>(0.20,0.59)	0<br>0.08<br>(0,0.46)	0<br>0<br>(0,0)

Table S1: Estimation of R and 95% credible intervals for human cases of H5N1 in Vietnam, vs. length of the infectious period γ^{-1}. In each case, the top row is the estimated value of R with maximum probability, the middle row is the mean R and the bottom row shows the limits of the credible interval. Note that for $R < 1$ longer infectious periods lead to *lower* estimates, see text.

4 Epidemiological Estimation from Stochastic Time Series

Here we address systematically the effects of different choices of the infectious period on the estimation of R. We also discuss the use of different assumptions for the discrete probability distribution of new cases in the estimation procedure, and derive the circumstances when Poisson is adequate or instead when consideration of the negative binomial (with overdispersion) may be necessary. For completeness, we also produce a new version of Table 1 (main text) containing R bounds for H5N1 influenza in humans for Vietnam using the negative binomial distribution.

4.1 Duration of the infectious period

The duration of the infectious period γ^{-1} is usually estimated from direct patient observation. That the estimate of R can depend on γ^{-1} is well known in the context of standard SIR models and has been discussed elsewhere [4, 5, 10]. For example in Figure S4 we show the evolution of the estimated R during an epidemic season of H3N2 with $\gamma = (2.6)^{-1}$ days^{-1} as in [4] and $\gamma = (4.1)^{-1}$ days^{-1} as in [1]. Table S1 shows the estimates of R for confirmed cases of H5N1 avian influenza in humans in Vietnam for a range of physiologically meaningful infectious periods, and different θ, as in the main text.

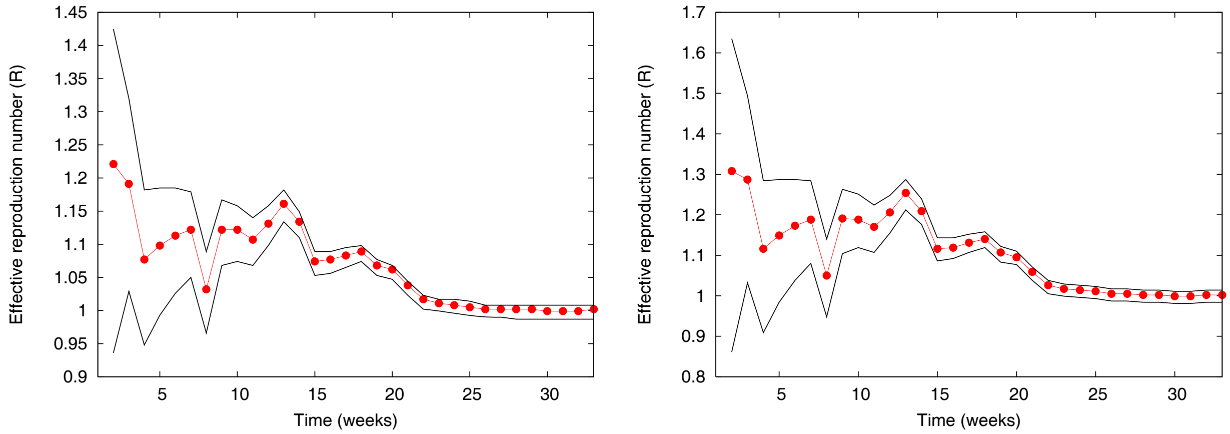

Figure S4: Sequential estimation of R for seasonal H3N2 influenza in 2004-05 in the USA, based on the number of new weekly isolates. $R = 1.22,\ 1.31$ at the maximum, before susceptible depletion, for $\gamma^{-1} = 2.6$ (left) and $\gamma^{-1} = 4.1$ (right), respectively. The corresponding estimate for $\gamma^{-1} = 7$ days (not shown) is $R = 1.43$.

Observation of Table S1 and of Figure S4 indicates that longer infectious periods lead to larger estimates of R, if $R > 1$, as is well known [4, 5, 10]. However, if $R < 1$ then longer infectious periods lead instead to *smaller* estimates of R. To understand this effect consider that the data remains invariant, so that the rate of change must remain constant under changes in γ, i.e. for any two γ_a, γ_b we must have

$$\frac{R^a - 1}{R^b - 1} = \frac{\gamma_b}{\gamma_a}, \tag{1}$$

where we neglected the usual $S(t)/N$, for simplicity - its inclusion does not change the discussion. Now assume e.g. that $\gamma_a^{-1} > \gamma_b^{-1}$, so that we obtain the relations

$$R^a > R^b \quad \text{and} \quad R^b > 1, \qquad \text{or} \quad R^a < R^b \quad \text{and} \quad R^b < 1, \tag{2}$$

which demonstrate the general effect of changing γ^{-1} on the estimates of R.

4.2 Different discrete distributions: overdispertion and R estimation

The Poisson distribution is the most widely used of all discrete distributions because it requires the fewest number of assumptions about the nature of a discrete stochastic process. It is the

maximal entropy distribution describing a discrete process occurring with a given mean λ, but that is otherwise random and uncorrelated in time. The Poisson distribution however predicts that the average of its random variable equals the variance (and both are given by λ), a feature that is sometimes violated by real data [9].

In some specific circumstances [6, 7, 9] epidemic time series can show overdispersion. This can be readily modeled by substituting the Poisson distribution by another discrete probability density function with the desired properties, usually a negative binomial, denoted here by $NegBin(\lambda, \omega)$. The negative binomial is parameterized by two quantities: the mean λ as before, and $\omega > 0$ usually called the clumping (or overdispersion) parameter. Note that a larger variance does not in general affect the estimation of the mean R, but does affect its associated credible interval (see below). It may also play a role in sequential Bayesian updates. This increased functional freedom must in turn be informed by phenomenology, to determine the appropriate value of ω. We note that in the limit $\omega \to +\infty$ one recovers the Poisson distribution, whereas for $\omega = 1$ one obtains the geometric distribution. In this sense the negative binomial generalizes several other discrete distributions.

The association of the clumping parameter with new infectious cases $\omega = \Delta T(t)$, as in [6, 7], is often made leading to a coefficient of variation in the data $CV \sim 1/\sqrt{\Delta T(t)}$, so that the variance to mean is reduced as more cases occur, a familiar assumption that also makes possible the use of deterministic epidemic models in that regime.

With these assumptions it is interesting to ask how large must ω be for the Poisson distribution to be applicable and to investigate the interplay between estimating R and the magnitude of ω. To answer these questions consider that the variance of the negative binomial distribution is given by

$$\lambda \left(1 + \frac{\lambda}{\omega}\right), \tag{3}$$

and therefore exceeds that of the Poisson distribution by a multiplicative factor of $1 + \lambda/\omega$. For a standard SIR epidemic process at time t, $\lambda/\omega = 1 + \tau\gamma \left(R_0 \frac{S(t)}{N} - 1\right)$, which becomes particularly

Infectious period (days)	θ=1	θ=0.8	θ=0.6	θ=0.4	θ=0.2
2.6	0.82<br>0.83<br>(0.70,0.94)	0.70<br>0.70<br>(0.58,0.82)	0.59<br>0.59<br>(0.48,0.70)	0.49<br>0.49<br>(0.38,0.59)	0<br>0.34<br>(0,0.46)
4.1	0.72<br>0.72<br>(0.52,0.91)	0.61<br>0.60<br>(0.43,0.77)	0.51<br>0.51<br>(0.34,0.66)	0.41<br>0.41<br>(0.27,0.56)	0<br>0.02<br>(0,0.32)
7.0	0.52<br>0.52<br>(0.19,0.83)	0.46<br>0.46<br>(0.20,0.73)	0.40<br>0.40<br>(0.17,0.64)	0<br>0.30<br>(0,0.55)	0<br>0<br>(0,0)

Table S2: Estimates of R for the H5N1 human case Vietnam time series, see Figure 1a (main text). In each case, the top row is the estimated value of R with maximum probability, the middle row is the mean R and the bottom row shows the limits of the credible interval. Results are obtained via an estimation procedure analogous to that for Table S1, but using a negative binomial for the distribution of the number of new cases, instead of Poisson, with same mean and clumping parameter $\omega = \Delta T(t)$. Note that the estimates for R obtained via the Poisson and negative binomial distributions are mutually consistent, with the difference that confidence intervals are slightly wider for the latter.

simple with the choice $\gamma\tau = 1$, leading to

$$\frac{\lambda}{\omega} = R_0 \frac{S(t)}{N}. \tag{4}$$

This shows that the Poisson distribution is a good approximation to an epidemic process with $R << 1$, as is characteristic of emerging infectious diseases. It may also be a good approximation at the ending stages of an epidemic where $R_0 S(t)/N < 1$, even if $R_0 > 1$. On the other hand accounting for possible overdispersion may prove crucial in the beginning stages of an epidemic process, and assumes particular importance the larger $R > 1$. These considerations apply e.g. to measles outbreaks, which are characterized by $R_0 > 10$ [5, 6, 7]. They may also apply to seasonal influenza under special circumstances, for which R is large [8]. For completeness we give in Table S2 estimates for R and its 95% credible intervals obtained using a negative binomial distribution with $\omega = \Delta T(t)$ for Vietnam. As can be observed estimates of R obtained via the Poisson and negative binomial distributions are mutually consistent, with slightly wider confidence bounds obtained for the latter, as expected from its overdispersed character.

5 Further Applications: the 1918 influenza pandemic and anomaly detection in surveillance

Here we give further examples of the methodology, by applying it to data on excess mortality for the 1918 H1N1 influenza epidemic in selected cities in the USA, to show consistency of our estimates with previous work in the literature. Finally we include supplementary figures showing how the methodology can be used to predict future new cases and detect shifts in transmissibility, as discussed in the main text.

5.1 The 1918 Influenza epidemic in selected cities

Here we apply the methodology to the 1918 H1N1 influenza outbreak in selected cities, in order to gauge the consistency of our estimations with previous work in the literature. Figure S5 shows the estimated R for four selected cities in the USA, during the initial weeks of the 1918 influenza pandemic. Data is for excess mortality, obtained from Supplementary Information in [1].

Results are consistent with those reported in [1], although no explicit numerical values for R_0 are given there for each city. Moreover in Figure S6, we present the same graphs for an infectious period of $\gamma^{-1} = 4.1$ days as used in [4], and again obtain estimates of R_0 that are consistent with those reported in [4].

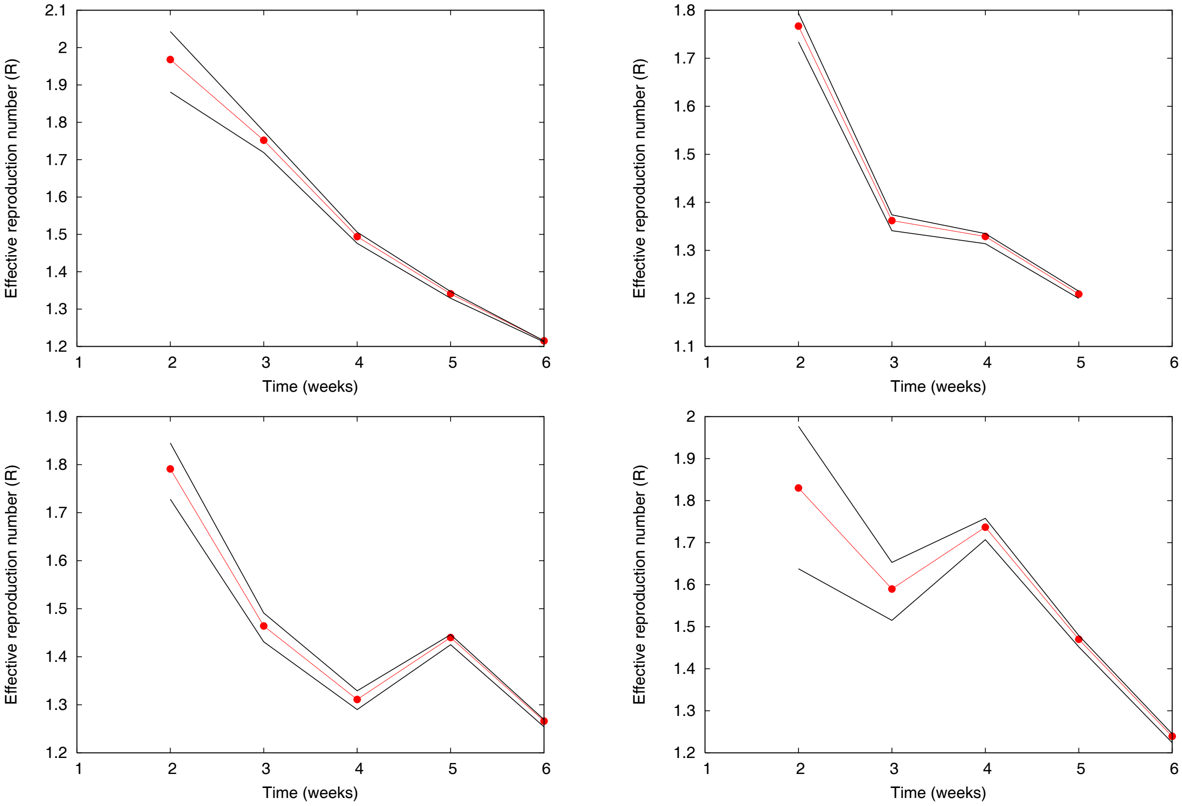

Figure S5: Evolution of R estimates over time (weeks) for the 1918 H1N1 influenza epidemic in four selected cities in the USA: New York, Los Angeles, Pittsburgh and Atlanta (left to right, top to bottom). The decay of R over time is due to the depletion of susceptibles. Data is for excess mortality, from Ref. [1]. Estimates are for an infectious period $\gamma^{-1} = 2.6$ days.

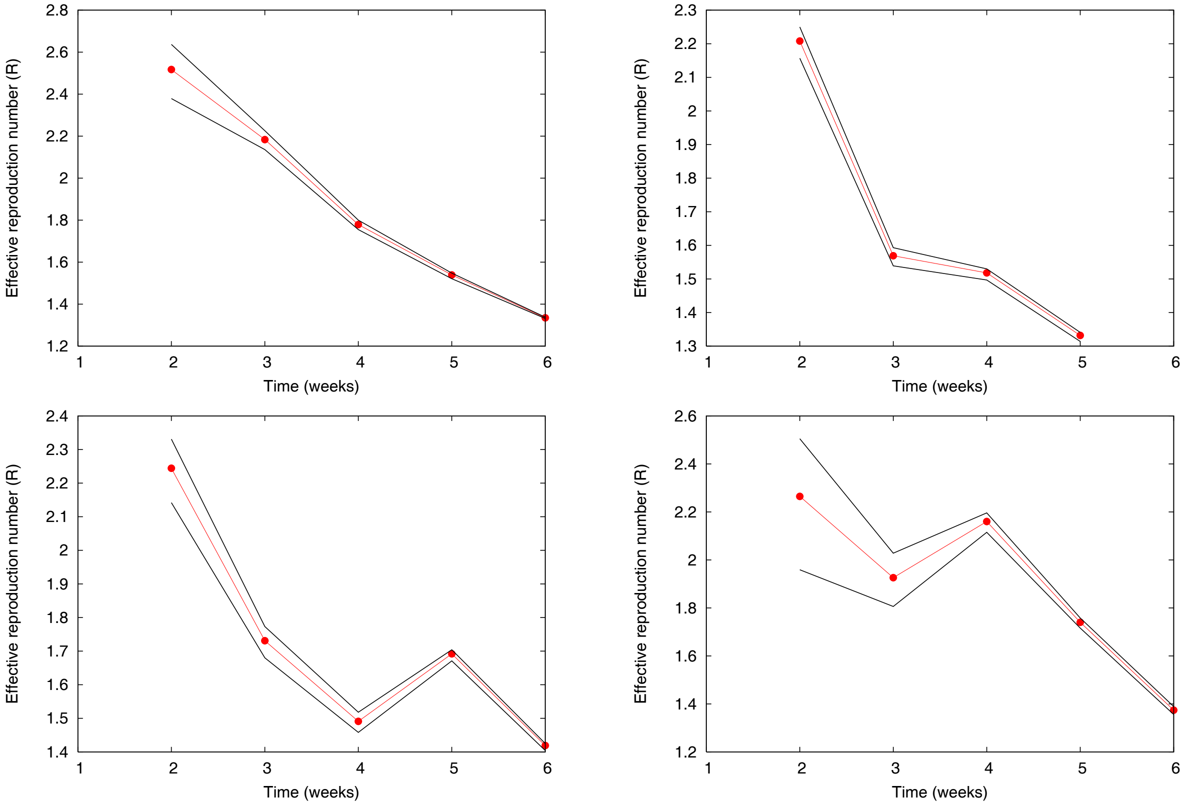

Figure S6: The same as Fig. S5 for an infectious period $\gamma^{-1} = 4.1$ days. Note that the evolution is identical in the two cases but that the estimate of the magnitude of R differs somewhat, with the longer infectious periods yielding larger R, see text.

5.2 Shifts in transmissibility as statistical anomalies vs. the magnitude of R

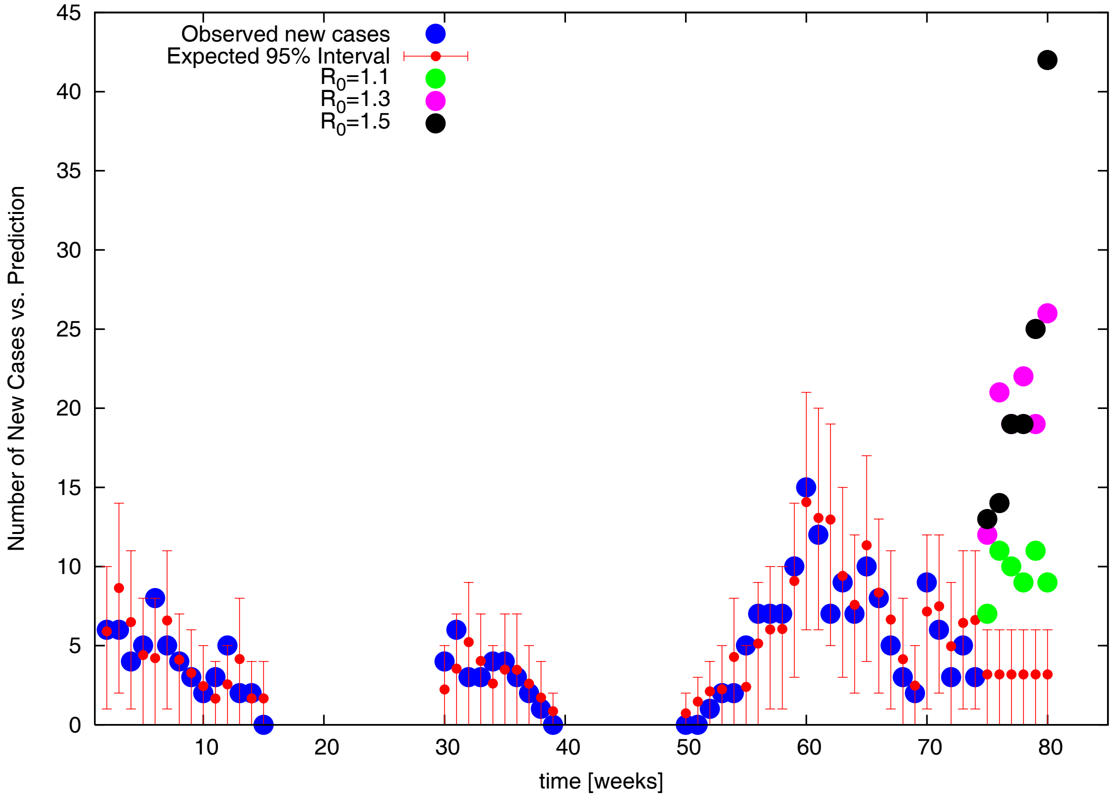

Figure S7: Prediction for new cases of avian influenza (simulated data $R_0 = 0.8$, with infections from reservoir taken from Vietnam time series, Fig. 1a of main text) vs. realized new cases (blue dots). Numbers of new cases that fall outside expected credible bounds indicate shifts in R, here from $R_0 = 0.8$ to $R_0 = 1.1$, 1.3 and 1.5 (green, purple and black dots), at week 75 and beyond. Although even shifts in R_0 to values just above 1 produce clear anomalies, larger values of R_0 lead to larger case numbers further away from the upper credible bound. The detection of such statistical anomalies can be used to trigger surveillance investigations and/or intervention measures. Anomaly detection can also flag potential surveillance errors, incorporating new cases into the iterative estimation procedure if they fall within bounds and rejecting them otherwise, as shown here during weeks 75-80. As shown here, persistent anomalies signal very likely changes in epidemiological conditions.

References

[1] Mills, C. E., Robins, J. M. & Lipsitch, M. Transmissibility of 1918 pandemic influenza. Nature 432, 904-906 (2004).

[2] Mills, C. E., Robins, J. M., Bergstrom, C. T. & Lipsitch, M. Pandemic Influenza: Risk of Multiple Introductions and the Need to Prepare for Them. PLoS Biol 3, e135 (2006).

[3] Antia, R., Regoes, R. R., Koella, J. C. & Bergstrom, C. T. The role of evolution in the emergence of infectious diseases. Nature 426, 658-61 (2003).

[4] Fergusson, N. M., Cummings, D. A., Cauchemez, S., Fraser, C., Riley, S., Meeyai, A., Iamsirithaworn, S., & Burke, D. S. Strategies for containing an emerging influenza pandemic in Southeast Asia. Nature 437, 209-214 (2005).

[5] Anderson, R. M. & May, R. M. Infectious diseases of humans (Oxford University Press, Oxford, 1995).

[6] Bjornstad, O. N., Finkenstädt, B. F. & Grenfell, B. T. Dynamics of measles epidemics: estimating scaling transmission rates using a time series SIR model. Ecological Monographs 72, 169-184 (2002).

[7] Grenfell, B. T., Bjornstad, O. N., & Finkenstädt, B. F. Dynamics of measles epidemics: Scaling noise, determinism, and predictability with the TSIR model. Ecological Monographs 72, 185-202 (2002).

[8] See e.g. Fraser, C., Riley, S., Anderson, R. M., & Fergusson, N. M. Factors that make an infectious disease outbreak controllable. PNAS 101, 6146-6151 (2004), and references therein.

[9] Kendall, D. G. Stochastic processes and population growth. Journal of Royal Statistical Society B 11, 230-264 (1949).

[10] Longini, I. M. Jr., Halloran, M. E., Nizam, A., Yang, Y. Containing pandemic influenza with antiviral agents. American Journal of Epidemiology 159: 623-633 (2004).
